# Model-Guided Design and Optimization of CPA Perfusion Protocols for Whole Organ Cryopreservation

**DOI:** 10.1007/s10439-023-03255-5

**Published:** 2023-06-23

**Authors:** Zonghu Han, Joseph Sushil Rao, Srivasupradha Ramesh, Jan Hergesell, Bat-Erdene Namsrai, Michael L. Etheridge, Erik B. Finger, John C. Bischof

**Affiliations:** 1 Department of Mechanical Engineering, University of Minnesota, Minneapolis, MN USA; 2 Department of Surgery, University of Minnesota, Minneapolis, MN USA; 3 Schulze Diabetes Institute, University of Minnesota, Minneapolis, MN USA; 4 Institute for Multiphase Processes (IMP), Leibniz University, Hannover, Germany; 5 Institute for Engineering in Medicine, University of Minnesota, Minneapolis, MN USA; 6 Department of Biomedical Engineering, University of Minnesota, Minneapolis, MN USA

**Keywords:** Organ perfusion optimization, Cryoprotectant, Toxicity, Transport, Organ vitrification

## Abstract

Vitrification could enable long-term organ preservation, but only after loading high-concentration, potentially toxic cryoprotective agents (CPAs) by perfusion. In this paper, we combine a two-compartment Krogh cylinder model with a toxicity cost function to theoretically optimize the loading of CPA (VMP) in rat kidneys as a model system. First, based on kidney perfusion experiments, we systematically derived the parameters for a CPA transport loading model, including the following: *V*_b_ = 86.0% (*r*_a_ = 3.86 μm), *L*_p_ = 1.5 × 10^–14^ m^3^/(N·s), *ω* = 7.0 × 10^–13^ mol/(N·s), *σ* = 0.10. Next, we measured the toxicity cost function model parameters as *α* = 3.12 and *β* = 9.39 × 10^–6^. Combining these models, we developed an improved kidney-loading protocol predicted to achieve vitrification while minimizing toxicity. The optimized protocol resulted in shorter exposure (25 min or 18.5% less) than the gold standard kidney-loading protocol for VMP, which had been developed based on decades of empirical practice. After testing both protocols on rat kidneys, we found comparable physical and biological outcomes. While we did not dramatically reduce toxicity, we did reduce the time. As our approach is now validated, it can be used on other organs lacking defined toxicity data to reduce CPA exposure time and provide a rapid path toward developing CPA perfusion protocols for other organs and CPAs.

**Supplementary Information:**

The online version contains supplementary material available at 10.1007/s10439-023-03255-5.

## Introduction

Ischemic injury to donor organs is a major challenge in transplantation. Maximum tolerable ischemic times with current organ preservation techniques are typically 4–6 h for heart and lung; 8–12 h for liver, intestine, and pancreas; and up to 36 h for kidney transplants [11], but in all cases shorter is better. Many transplantable organs are discarded when preservation times are prolonged, further exacerbating the ever-increasing imbalance between organ supply and demand.

Vitrification is a long-term preservation approach that could enable the storage of organs for years, which would revolutionize how organs are recovered, allocated, and utilized for transplantation. Avoidance of destructive ice formation during the vitrification of organs requires high concentrations (8–9 M) of cryoprotective agents (CPA), which can be toxic. Therefore, successful vitrification while maintaining high organ viability requires a balance between loading a high enough concentration of CPA to achieve vitrification while simultaneously limiting toxicity. This is a complex problem to address, as it requires accurately describing the mass transport in the tissue (both perfusion in and diffusion from the vasculature); osmotic effects at the cellular level; and time, temperature, and concentration-dependent CPA toxicity.

Successful vascular CPA perfusion protocols have been developed and optimized through years of primarily empirical testing. The first vitrification of a whole organ (rabbit kidney) was achieved by Fahy et al. in [7] and has been more recently demonstrated in additional organ systems [8, 12, 38, 39]. Decades of effort in the kidney has refined a general protocol that allows for successful loading and unloading with a functioning kidney at the end. We aim to build off the principles demonstrated through those efforts, and leverage established biological modeling concepts developed in cells and tissues, to demonstrate a general mathematical formulation that allows the systematic optimization of CPA perfusion in organs.

Historically, efforts to understand CPA loading and unloading and CPA toxicity have been conducted separately, as shown in Table 1. For instance, there are various models for CPA transport in biological organs, such as the Krogh model [37], multi-dimensional model [28], network thermodynamic model [26], and others. There are also several approaches to understanding CPA toxicity, such as the solution effect model [5, 30] (qv* model [9]), CPA interaction model [21], and toxicity cost function model [3]. However, to our knowledge, there has yet to be a comprehensive effort to simultaneously study these effects in organ systems. To address this limitation, we leveraged two well-established models in this paper: (i) the Krogh cylinder model, which predicts CPA transport and osmotic stress in tissue, and (ii) the toxicity cost function model, which describes the chemical toxicity kinetics.

**Table 1:** Examples of CPA transport and toxicity models

Transport models	
Krogh cylinder model [35, 37]	A representative repeating functional unit of the organ containing vascular and tissue compartments
Multi-dimensional model [28]	A porous media model applied to a multi-dimensional microvascular unit (e.g., hepatic acinus)
Network thermodynamic model [25]	A multi-compartmental model using a network thermodynamic model transport between interstitial and intracellular compartments
Toxicity models	
Solution effects model [30, 31]/qv* model [5, 9]	Toxicity caused by dehydration/concentrated salt in the solution
CPA toxicity models [1, 16–18, 23, 40, 41]	Different mechanisms of CPA toxicity include: ion channels blockage [17], cell and mitochondrial membrane alteration [41], cellular apoptosis [23], telomerase activity inhibition [40], affecting ATP production [18], protein glycosylation [16], cytoskeleton and mitotic spindle architecture disruption, and DNA denaturation [1]
CPA interaction models [21]	Beneficial component interactions in the CPA cocktail
Toxicity cost function model [2, 3]	The cumulative toxicity incurred during CPA exposure in cells and 3D tissues

The Krogh cylinder model was selected because of its experimentally validated feasibility and simplicity [35] and because it allows direct experimental measurement of parameters which can then be used to estimate CPA diffusion and concentration inside the organ. August Krogh first proposed the Krogh cylinder model in 1919 to study oxygen transport from capillaries to tissues [24]. It was then applied by Rubinsky et al. to study the CPA transport kinetics inside organs in 1982 [37]. A basic assumption in the model is that the mass transfer from the vasculature into the tissue cells occurs primarily at the capillary level, instead of larger vessels, since the surface-area-to-volume ratio of capillaries is much larger than for all other vessels [37]. Therefore, the vascularized organ is represented by many identical cylindrical or hexagonal prism units, each of which consists of a central capillary surrounded by a volume which represents cells of the tissue. The mass transfer in an organ is analyzed by considering the processes in such a unit to be typical and representative of the processes in the whole organ [37]. Inside the Krogh cylinder, the kinetics of water and CPA transport happening at the capillary membrane are described by Kedem–Katchalsky (KK) Eqs.  [22], which are derived from irreversible thermodynamics, shown as follows:

1 $${J_{\text{v}}} = S \cdot {L_{\text{p}}}[({P_{\text{f}}} - {P_{\text{t}}}) - {R_{\text{g}}}T({C_{\text{is,f}}} - {C_{\text{is,t}}} + \sigma \{ {C_{\text{cpa,f}}} - {C_{\text{cpa,t}}}\} )],$$

2 $${J_{{\text{cpa}}}} = S[\omega {R_{\text{g}}}T({C_{\text{cpa,f}}} - {C_{\text{cpa,t}}})] + {J_{\text{v}}}(1 - \sigma )\left( {\frac{{{C_{\text{cpa,f}}} + {C_{\text{cpa,t}}}}}{2}} \right),$$

where *J*_v_ is the total volumetric flow rate (water and CPA) through the capillary membrane (m^3^/s), *J*_cpa_ is the CPA flow rate (mol/s), *S* is the surface area of the membrane (m^2^), *L*_p_ is the hydraulic conductivity [m^3^/(N·s)], *P* is the hydraulic pressure (N/m^2^), *R*_g_ is the universal gas constant [J/(mol·K)], *T* is the temperature (K), *C* is the concentration (mol/m^3^), *ω* is the CPA permeability [mol/(N·s)], and *σ* is the reflection coefficient. With regard to the subscripts for concentration, *t* and *f* refer to the concentration in the tissue and the concentration of the fluid flowing in the capillary, respectively; and *cpa* and *is* refer to the total CPA concentration and then the concentration of just the impermeable solutes (e.g., sugars, sugar alcohols, and polymers), respectively.

Exposure to high CPA concentrations causes injury to tissues by two mechanisms: (1) mechanical (osmotic) damage and (2) chemical (toxicity) damage. Osmotic damage can be reduced by controlling the rates at which CPA is added and removed to prevent excessive volume excursions caused by transient intra- versus extracellular concentration differences. Chemical toxicity is largely determined by the choice of CPA. Still, it can be minimized by carefully designing the loading and removal protocol to reduce exposure time at higher concentrations while still allowing the biological system to equilibrate to the CPA concentrations necessary for vitrification. The CPA we used in this paper was VMP (8.4 M), which contains DMSO, formamide, ethylene glycol (EG), X-1000, and Z-1000 (these last two are considered “ice blockers”), which has proven to have an acceptable toxicity profile in kidneys [9].

The toxicity cost function model, which explores CPA toxicity in cells or tissues as a function of exposure concentration, time, and temperature, is the only model that studies the cumulative CPA toxicity and was selected for further study here. Benson et al. analyzed historical data, developed a power law relationship between the toxicity rate and CPA concentration at a constant temperature, and proposed using a toxicity cost function, *J*_tox_ [3].

3 $$\left\{ {\begin{array}{*{20}{l}} {\begin{array}{*{20}{l}} {k = \beta \cdot C_{\text{cpa,t}}^\alpha } \\ {\frac{{{\text{d}}N}}{{{\text{d}}t}} = - k \cdot N} \\ {{J_{{\text{tox}}}} = \int_0^{t_f} {k\;{\text{d}}t = \int_0^{t_f} {\beta \cdot C_{\text{cpa,t}}^\alpha \;{\text{d}}t} } } \end{array}} \\ {\frac{N}{{N_0}} = \exp ( - {J_{{\text{tox}}}})} \end{array}} \right.,$$

where *k* is the toxicity rate (1/min), *α* and *β* are two constants that depend on the CPA formulation and its interaction with a specific biological system and the temperature, *C*_cpa,t_ is the CPA concentration inside the tissue, *N* is the viability after the exposure time, *t*_f_ is the duration of CPA exposure, *N*_0_ is the initial viability, and *J*_tox_ is the toxicity cost function which is a relative toxicity measure that describes the cumulative (time-integral) toxicity during CPA exposure.

In this paper, we were able to, for the first time, (1) combine the transport and toxicity kinetics models in whole organs, (2) systematically derive the parameters in the transport model and determine their values using perfusion, and (3) apply the combined model to optimize kidney perfusion loading with CPA. Finally, this work provides direct validation of the modeling approach in the rat kidney, allowing it to be further applied to enable vitrification without toxicity in other organ systems. In short, this work has the potential to enable cryopreservation protocols through vitrification to be more broadly applied across organs used in transplant medicine.

## Materials and Methods

### Mathematical Model

**Fig. 1:**
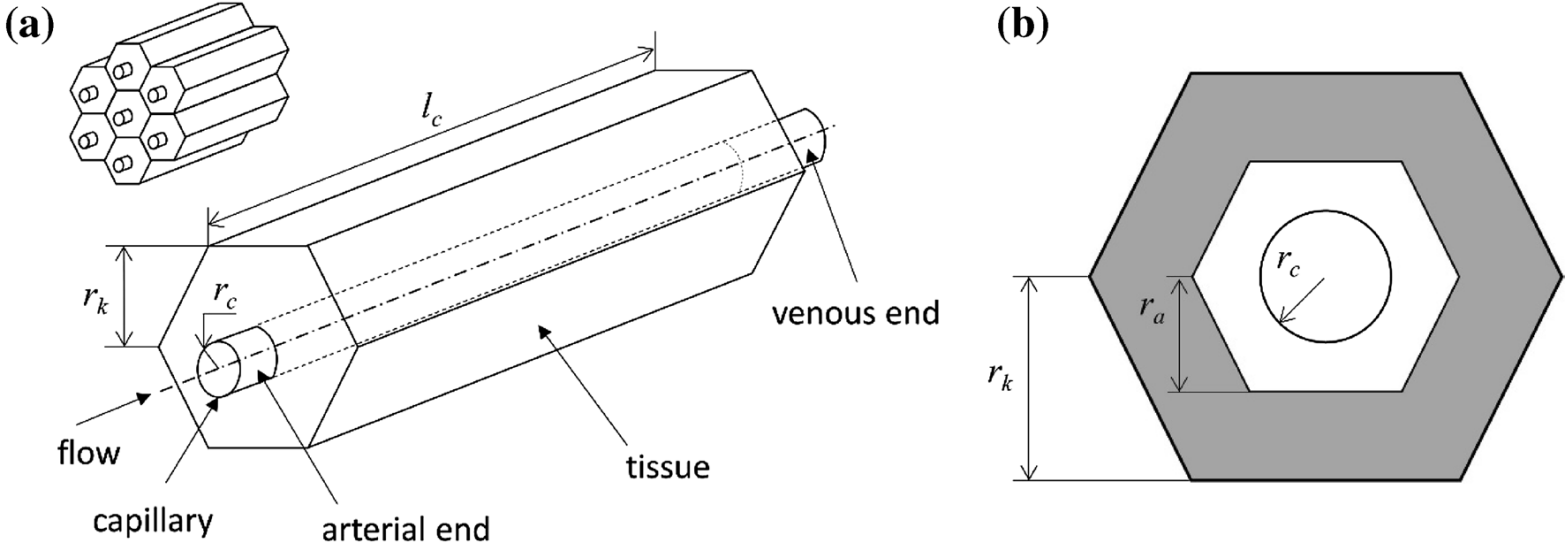
Krogh model geometry. **a** Krogh cylinder stacks and each unit with description of relevant parameters. **b** Cross section view of the Krogh cylinder, *r*_k_ is the apothem of the hexagonal prism (Krogh unit), *r*_a_ is the apothem of the hexagonal prism of the active volume, and *r*_c_ is the radius of the capillary. The gray area refers to the non-solvent (or solid) inactive volume

In the Krogh cylinder model, the outer boundary is fixed due to the symmetrical condition, and the radius of the vessel changes due to water and CPA transport (i.e., characteristic “shrink-swell” in the surrounding tissue). As shown in Fig. 1, the geometric parameters consist of *r*_c_ (capillary radius), *r*_k_ (= ∆*x*/2), half-length of the intercapillary distance (∆*x*), which also equals the apothem of the hexagon, and *l*_c_ (capillary length). Rat capillary diameters (*d*_c_) were reported ranging from 4 to 8 μm [19, 27, 29, 33], capillary lengths (*l*_c_) were reported ranging from 55 to 58 μm [33, 34], and intercapillary distance (∆*x*) is ~ 15 μm [27]. In this paper, we use the mean of these values leading to *r*_c_ = 3 μm, *r*_k_ = 7.5 μm, and *l*_c_ = 55 μm. Based on dimensionless analysis, it can be assumed that the concentration in the fluid and in the tissue can be taken to be radially and axially independent [37].

Another geometric parameter is the inactive volume fraction, *V*_b_, which is the tissue volume fraction that does not participate in osmotic activities (i.e., non-solvent). That is, the dry or solid volume after desiccation [32, 36]. This value is determined in small-scale samples by exposing the sample to various solutions with different osmolarities. Based on the observed volumetric changes and the Boyle van’t Hoff relationship, *V*_b_ can be determined. However, no one has determined *V*_b_ for whole organs, since the organ consists of not only cells but also extracellular spaces, making it a complex process to observe and describe. Therefore, *V*_b_ for cells cannot be directly applied to organ perfusion kinetics. In this paper, we derived, for the first time, the organ Boyle van’t Hoff equation and determined the inactive volume fraction using perfusion.

Based on the assumption that the organ is composed of a series of identical Krogh cylinder/prism units in parallel, the vascular resistance in the organ can be represented by

4 $$\left\{ {\begin{array}{*{20}{l}} {\Delta P = \Delta {P_1} = \Delta {P_2} = ...} \\ {Q = n \cdot q} \\ {{R_{{\text{single}}}} = \frac{\Delta P}{{\mu \cdot q}} = \frac{\Delta P}{{\mu \cdot Q/n}} = nR} \end{array}} \right.,$$

where *∆P* is the pressure difference between the inlet and outlet (Pa), *n* is the number of Krogh units in parallel inside an organ, *Q* is the total flow rate (m^3^/s), *q* is the flow rate of each Krogh unit (m^3^/s), *R* is the total vascular structural resistance of the kidney (1/m^3^), *R*_single_ is the vascular resistance of a single Krogh unit (1/m^3^), and *μ* is the viscosity of the perfused solution (Pa·s). Both *∆P* and *Q* can be obtained directly from perfusion data, and *R* can be calculated. However, *R*_single_ is not always available since *n* is an organ-dependent variable. Note that the vascular structural resistance, *R* (= *∆P*/*μQ*), should be distinguished from the perfusion resistance, *R*_p_ (= *∆P*/*Q*), where *R* directly reflects the resistance due to the vascular structural (vascular diameter) and *R*_p_ additionally reflects the viscosity and is mostly used in practice.

Inside each Krogh cylinder unit, the flow is assumed to be laminar (due to the small capillary size) and therefore governed by Poiseuille’s Eq.  [35]:

5 $$q = \frac{{\pi r_{\text{c}}^4 \cdot \Delta P}}{{8\mu \cdot {l_{\text{c}}}}},$$

where *r*_c_ is the radius of the capillary inside each Krogh cylinder unit and *l*_c_ is the length of the Krogh unit. If we rearrange Eq. (5) and substitute the relationship of *Q* = *nq*, we can get

6 $$\frac{\Delta P}{{Q \cdot \mu }} = R = \frac{{8{l_{\text{c}}}}}{{n\pi r_{\text{c}}^4}}.$$

For a Krogh unit, the parameter *r*_k_ is half of the interpapillary distance, which is also the apothem of the hexagonal prism. However, both the tissue and cell have an inactive volume, leading to a total effective, inactive volume for the Krogh unit. To simplify the calculation, we can assume the active volume effectively creates a smaller hexagonal prism, accounting for the active volume only with an apothem of *r*_a_. The relationship between *V*_b_ and* r*_a_ is

7 $${V_{\text{b}}} = \frac{{2\sqrt 3 (r_{\text{k}}^2 - r_{\text{a}}^2)}}{{2\sqrt 3 \;r_{\text{k}}^2 - \pi r_{{\text{c0}}}^2}}.$$

The initial active volume is

8 $${V_0} = {l_{\text{c}}} \cdot (2\sqrt 3 \cdot r_{\text{a}}^2 - \pi r_{{\text{c0}}}^2),$$

where* r*_c0_ is the initial capillary radius under isotonic conditions. When the kidney is perfused with a non-isotonic solution, the volume of the cylinder will shrink or swell to maintain osmotic balance, leading to a change in capillary radius. If we are perfusing a solution with an osmolarity of *M*, then the equilibrated active volume at the time *t* is

9 $$V(t) = \frac{{{M_{{\text{iso}}}}}}{M}{V_0},$$

where *M*_iso_ is the isotonic osmolarity (290 mOsm). According to the geometric relationship, we have

10 $${r_{\text{c}}}(t) = \sqrt {\frac{{2\sqrt 3 r_{\text{a}}^2 - V(t)/{l_{\text{c}}}}}{\pi }} ,$$

where *r*_c_(*t*) is the capillary radius at time *t*.

Substituting Eqs. (10), (9), and (8) into Eq. (6), we get

11 $$R = \frac{{8\pi {l_{\text{c}}}}}{n} \cdot \frac{1}{{{{[2\sqrt 3 r_{\text{a}}^2 - \frac{{{M_{{\text{iso}}}}}}{M}(2\sqrt 3 r_{\text{a}}^2 - \pi r_{{\text{c0}}}^2)]}^2}}}.$$

Rearranging Eq. (11), we get the following equation, which we call the organ Boyle van’t Hoff equation:

12 $$\frac{1}{\sqrt R } = - \sqrt {\frac{n}{8l\pi }} \cdot (2\sqrt 3 r_{\text{a}}^3 - \pi r_{{\text{c0}}}^2) \cdot \frac{{{M_{{\text{iso}}}}}}{M} + \sqrt {\frac{n}{8l\pi }} \cdot 2\sqrt 3 \cdot r_{\text{a}}^2.$$

On the right-hand side of the equation, for the same organ (*n* is constant), the second term is a constant, and the first term is directly proportional to 1/*M*. By plotting 1/$\sqrt{R}$ against *M*_iso_/*M*, we can obtain the slope and the *y*-intercept for the best-fitting curve. One example is shown in Fig. 3a. By dividing the slope by the *y*-intercept, we have

13 $$\frac{{{\text{slope}}}}{{y - {\text{intercept}}}} = - \frac{{2\sqrt 3 \cdot r_a^2 - \pi r_{{\text{c0}}}^2}}{{2\sqrt 3 \cdot r_{\text{a}}^2}}.$$

Then we can get the apothem of the active volume:

14 $${r_{\text{a}}} = \sqrt {\frac{{\pi r_{{\text{c0}}}^2}}{{2\sqrt 3 \cdot \frac{{{\text{slope}}}}{{y - \operatorname{intercept} }} + 2\sqrt 3 }}} .$$

Similar to *r*_a_, the other parameters in the transport model, *L*_p_, *ω*, and *σ*, have been widely studied on small-scale samples but not for the whole organ. Therefore, for the first time, we determined these parameters based on perfusion data in rat kidneys.

### CPA Preparation

All the carrier and CPA solutions were prepared as previously described. Three carrier solutions, Euro-Collins (EC), LM5, and LM5-XZ [LM5 + 1% X-1000 (w/v) and 1% Z-1000 (w/v)], were used in this study [9, 39]. EC, LM5, and LM5-XZ have osmolarities of 360, 283, and 330, respectively. EC and LM5 were used for determining the perfusion parameters, and LM5-XZ was used for CPA perfusion. VMP (8.4 M) was the only CPA used in this study [10]. The polymers X-1000 and Z-1000, needed for preparing VMP and LM5-XZ, were purchased (Twenty-first century Medicine, Fontana, CA).

### Perfusion of Kidneys

**Fig. 2:**
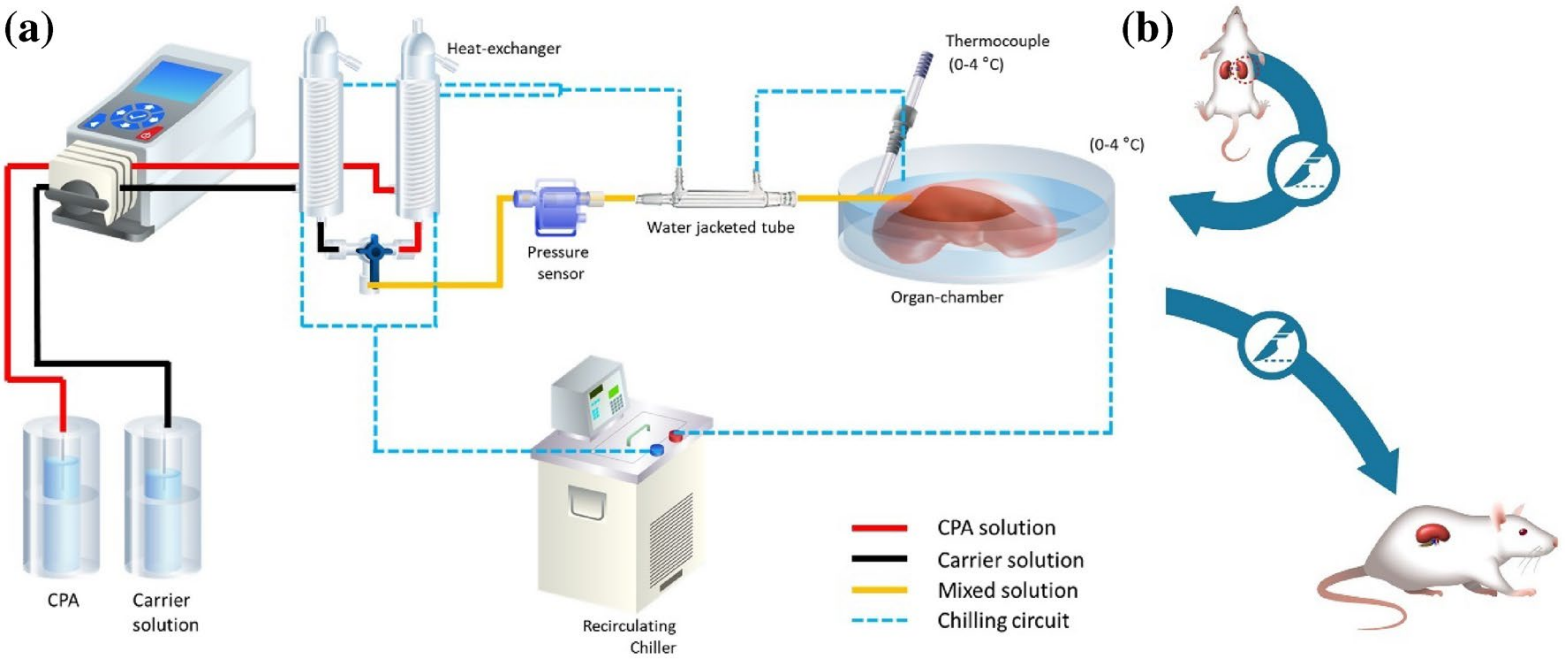
Schematic diagram of the **a** hypothermic perfusion circuit and **b** non-survival acute transplant model

All animal experiments were approved by the Institutional Animal Care and Use Committee (IACUC) at the University of Minnesota (IACUC Protocol: 2204-39970A). The surgical protocol for cannulation and nephrectomy has been described previously [39]. The rat kidneys were connected to a pressure- and flow-controlled multi-thermic perfusion system, as shown in Fig. 2a. The perfusion solutions were maintained between 0 and 4 °C, and the pressure was kept at 40 mm Hg except for the last step of VMP loading at 8.4 M. After 20-min of flushing, the concentration of VMP was increased in a ramp manner to a specific value and held constant for a few minutes. Then the concentration was stepped to 8.4 M and held while the pressure was simultaneously increased to 60 mm Hg.

### ***L***_p_, ***ω***, ***σ ***, and ***r***_a_ Determination by Perfusion

**Fig. 3:**
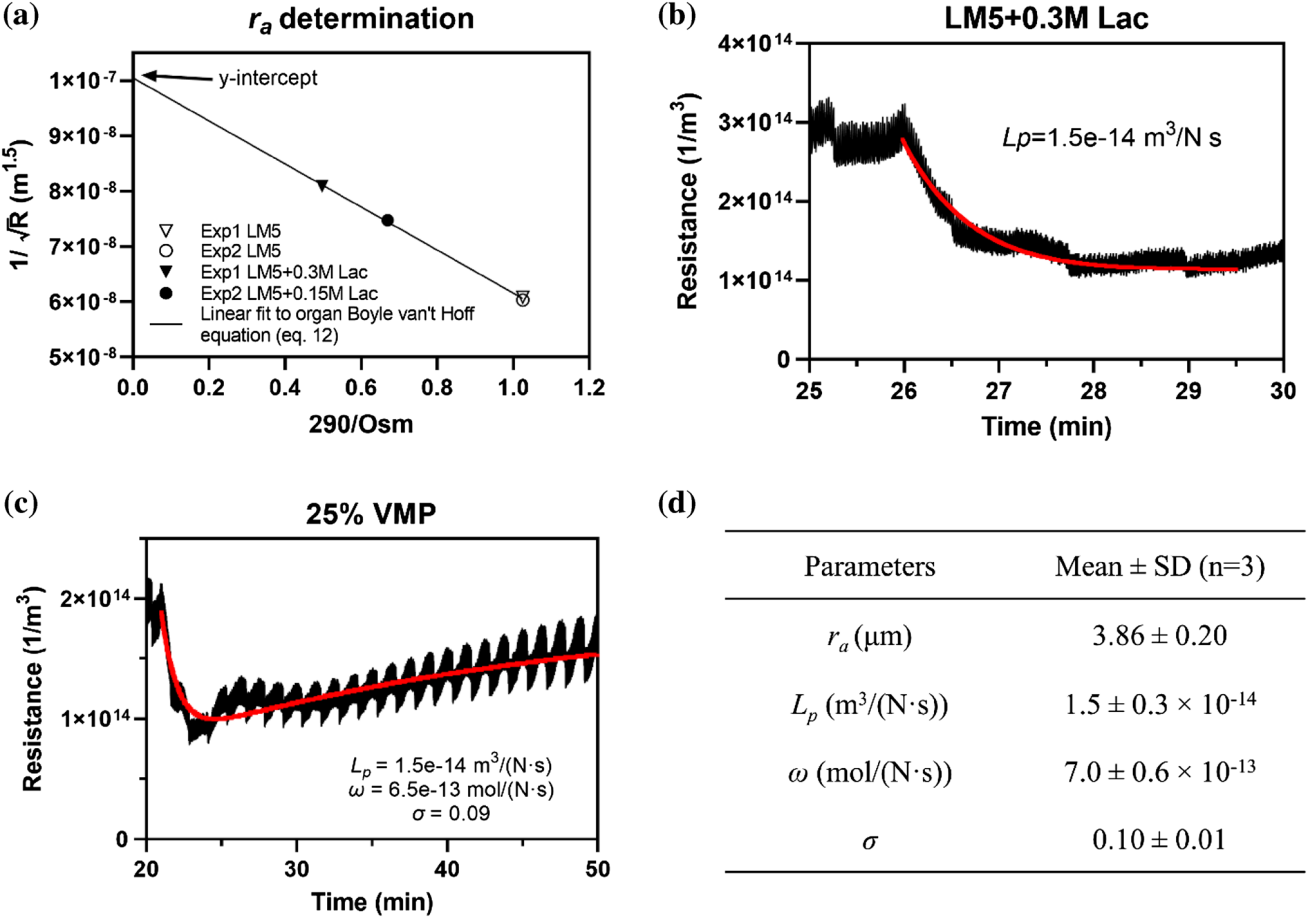
Parameters measured by kidney perfusion. **a** For *r*_a_, the kidney was perfused with LM5, LM5 + 150 mM lactose, and LM5 + 300 mM lactose solutions, and *r*_a_ was fit based on Eq. (12). **b** For *L*_p_, the kidney was perfused with LM5 and LM5 + 300 mM lactose solutions, and *L*_p_ was fit based on Eq. (1). **c** For *ω* and *σ*, the kidney was perfused with LM5 and 25% VMP solutions, and ω and σ were fit based on Eq. (2). **d** Summary table of the measured parameter values (mean ± standard deviation). Each parameter was determined from *n* = 3 separate kidney perfusion experiments

For *r*_a_ determination, the same kidneys were perfused with carrier solutions containing different concentrations of lactose, and the stabilized resistances were plotted against the osmolarities, as shown in Fig. 3a. A fitting curve was then used to calculate the apothem of the active volume, *r*_a_. For *L*_p_ determination, the kidneys were flushed with carrier solutions for 20–25 min to reach the stabilized baseline resistance, then perfused with carrier solution + 300 mM lactose and held for 15 min. Similarly, for the determination of *ω* and *σ*, the kidneys were flushed with LM5 and then perfused with 25% VMP for 30 min. The values of *L*_p_, *ω*, and *σ* were then obtained by fitting the model to the experimental curves, where *R*^2^ values were calculated and used to determine the quality of the fitting. All the models are coded in MATLAB R2020b (MathWorks).

### Micro-CT Imaging

The samples were scanned in a μCT imaging system (NIKON XT H 225, Nikon Metrology, MI). The setup and analysis have been previously described [14].

### Slice Preparation

Kidney slices were prepared by taking 5 mm punch biopsies from the renal cortex (approximated as representative of the whole kidney [10]), embedding in 3% (w/v) low melting agarose (IBI Scientific) diluted using PBS, sliced at 300 µm thickness using a Compresstome VF-310-0Z tissue vibratome (Precisionary Instruments), and stored in cold University of Wisconsin solution until use.

### Toxicity Testing Procedure

The slices were tested for baseline mitochondrial activity using a non-destructive alamarBlue (aB) assay [14], where each slice was placed in the mixed solution of 450 μl of DMEM media and 50 μl aB in an incubator for 50 min at 37 °C and 5% CO_2_/95% air. Fluorescence was measured on a plate reader (Synergy HT, BioTek) at 590 nm. The background fluorescence (i.e., that of media + alamarBlue alone) was subtracted from each measurement. Step loading and removal (0, 1.05 M, 2.1 M, 4.2 M, 6.3 M, and 8.4 M) were applied to reach the testing concentrations while minimizing osmotic stress. The time for each intermediate step to reach the target concentration is taken as 4 min since the calculated characteristic diffusion time is 173 s based on the equation *t*_char_ = *l*^2^/(2*D*), where *l* is the characteristic length of the slice (half-thickness = 150 μm) and *D* is the diffusivity (value was taken as 6.5 × 10^–11^ m^2^/s) [14]. The slices were then kept at the final concentration of VMP for 15, 30, 45, 60, 90, and 120 min to assess toxicity as a function of exposure time. After exposure, the slices were unloaded with decreasing steps at the reverse increments of loading. The slices were then placed in 24-well plates with 500 μl of DMEM media in an incubator for 50 min to rest the slices and for injury to become apparent. The alamarBlue assay was again performed on the slices after resting for 50 min and compared to the baseline values to obtain the viabilities, which are then normalized to the control group (cold storage in UW solution) for the reported values. The drop in alamarBlue readings was taken to indicate the decrease in viability due to toxicity induced in the slices by exposing them to VMP.

### Non-survival Acute Transplant Model

Male Lewis rats weighing 350–500 g were used as recipients to study the function of CPA-perfused kidneys. Recipient anesthesia was induced using 4% isoflurane and maintained with 1.5% isoflurane and 1 liter-per-minute oxygen via nose cone. Similar to the donor surgery, a cruciate abdominal incision was performed, and the infrarenal aorta and retroperitoneum were exposed. Bilateral renal artery and veins were ligated and divided and recipient kidneys were explanted. The artery and veins of CPA-perfused kidney were then prepared for an orthotopic transplant. The arterial and venous anastomoses were performed as previously described [13]. The ureter was cannulated using a PE-10-100 polyethylene tubing (0.011 ID X 0.025 OD; SAI Infusion Technologies), and urine output was measured in an Eppendorf tube. Venous blood samples were obtained from the inferior vena cava using a 31G hypodermic needle and 1 ml syringe every 15 min following reperfusion over 2 h. Blood gas analysis was assessed for all samples (ABL90 FLEX Plus, Radiometer, Brea, CA). Serum samples were used for creatinine assessments (Rat Creatinine Kit #80340, Crystal Chem, IL). At the end of perfusion, the kidney was flushed with normal saline and stored in 2% buffered neutral formalin for subsequent histologic processing.

### Statistical Analysis

Statistical analysis was performed in GraphPad Prism (GraphPad Software, Inc.). The number of biological replicates is indicated in each figure legend. All measurements represent distinct biological replicates taken from individual kidneys. For group comparisons, *t* test was used to determine statistical differences. A *P* value of < 0.05 was considered significant.

## Results

In order to develop a mass transport model for the CPA delivery in renal tissue, we began by determining the geometrical and transport kinetic parameters in the model. The apothem of the active volume cylinder, *r*_a_, was determined first. The same kidney was perfused with various solutions of differing osmolarity. One example is shown in Fig. 3a. Two sequential perfusions were performed on this kidney. In the first perfusion, it was perfused with LM5 and then LM5 + 150 mM lactose solution and in the second perfusion it was perfused with LM5 and then LM5 + 300 mM lactose solution. The resistance values were calculated using the steady-state flow rates at the four perfusion stages. We then plotted the parameters as suggested in Eq. (12) to obtain the best-fitting curve, which in turn validated the model-derived theoretical relationship between the resistance and the perfusate osmolarity. The determined average *r*_a_ value was 3.86 μm. For the *L*_p_ determination experiments, as shown in Fig. 3b, the kidney was perfused with LM5 first and then with LM5 + 300 mM lactose. The real-time resistance was plotted with respect to the perfusion time, and the best-fitted *L*_p_ for this kidney was 1.5 × 10^–14^ m^3^/(N·s). Similarly, the experiments to determine the CPA permeability and reflection coefficient, *ω* and *σ*, were performed by flushing the kidney with LM5 and then perfusing it with 25% VMP solution. The averaged fitted values for *ω* and *σ* were 7.0 × 10^–13^ mol/(N·s) and 0.10, respectively.

**Fig. 4:**
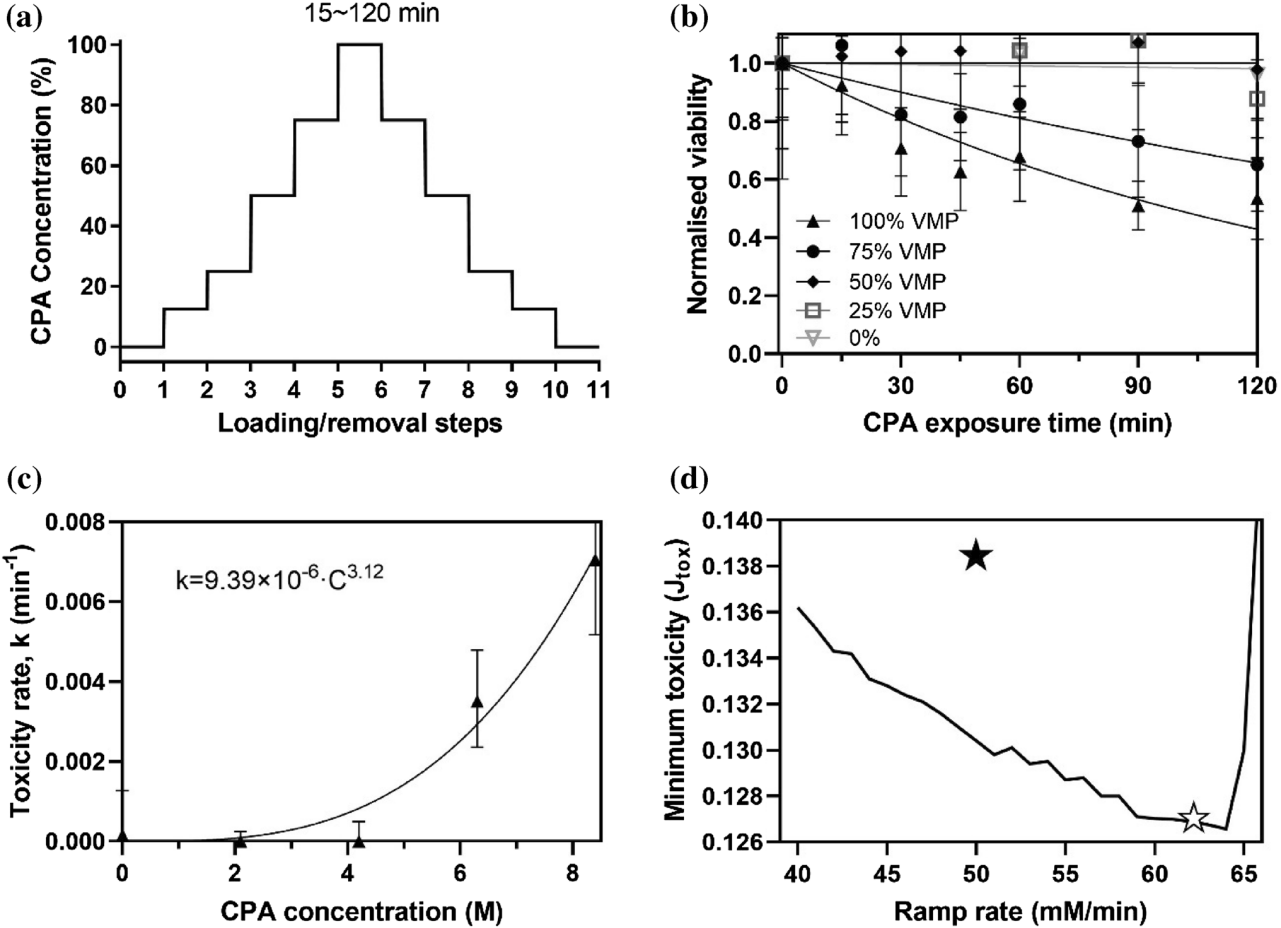
Toxicity cost function measurements using kidney slices and the results of kidney loading protocol optimization. **a** The loading and unloading protocol for kidney slices is shown. This was used to measure the toxicity rate at the testing concentration, e.g., 100% VMP. The time interval is 4 min for each step to reach the final testing concentration (e.g., 25 to 100%), and the exposure time at the testing concentration ranges from 15 to 120 min. **b** Viability of the kidney slices after 15–120-min exposure at different testing concentrations is shown (note: assumption is that toxicity is mainly from final testing concentration). *n* = 4 for each concentration at each exposure time. **c**) The toxicity rate as a function of the concentration of VMP was fit based on experimental data shown in **b** using Eq. (3). **d** Model-predicted minimum toxicity cost function values at each ramp rate were calculated. Solid and hollow stars represent the toxicity of the existing and optimized protocols, respectively. For ramp rates above ~ 64 mM/min, the ramp duration is limited due to increased osmotic stress, which subsequently requires a substantially longer “plateau” region at higher CPA concentrations, which produces a rapid increase in the minimum predicted toxicity (see Figures S2–4 in Supplementary material)

As shown in Fig. 4b, we used the exponential relationship to fit the viability data and obtained the best-fitting toxicity rate, *k*, at each concentration. We then plotted the toxicity rate, *k*, as a function of the VMP concentration in Fig. 4c. The best-fit parameters are *α* = 3.12 and *β* = 9.39 × 10^–6^ for VMP at 4 °C.

**Fig. 5:**
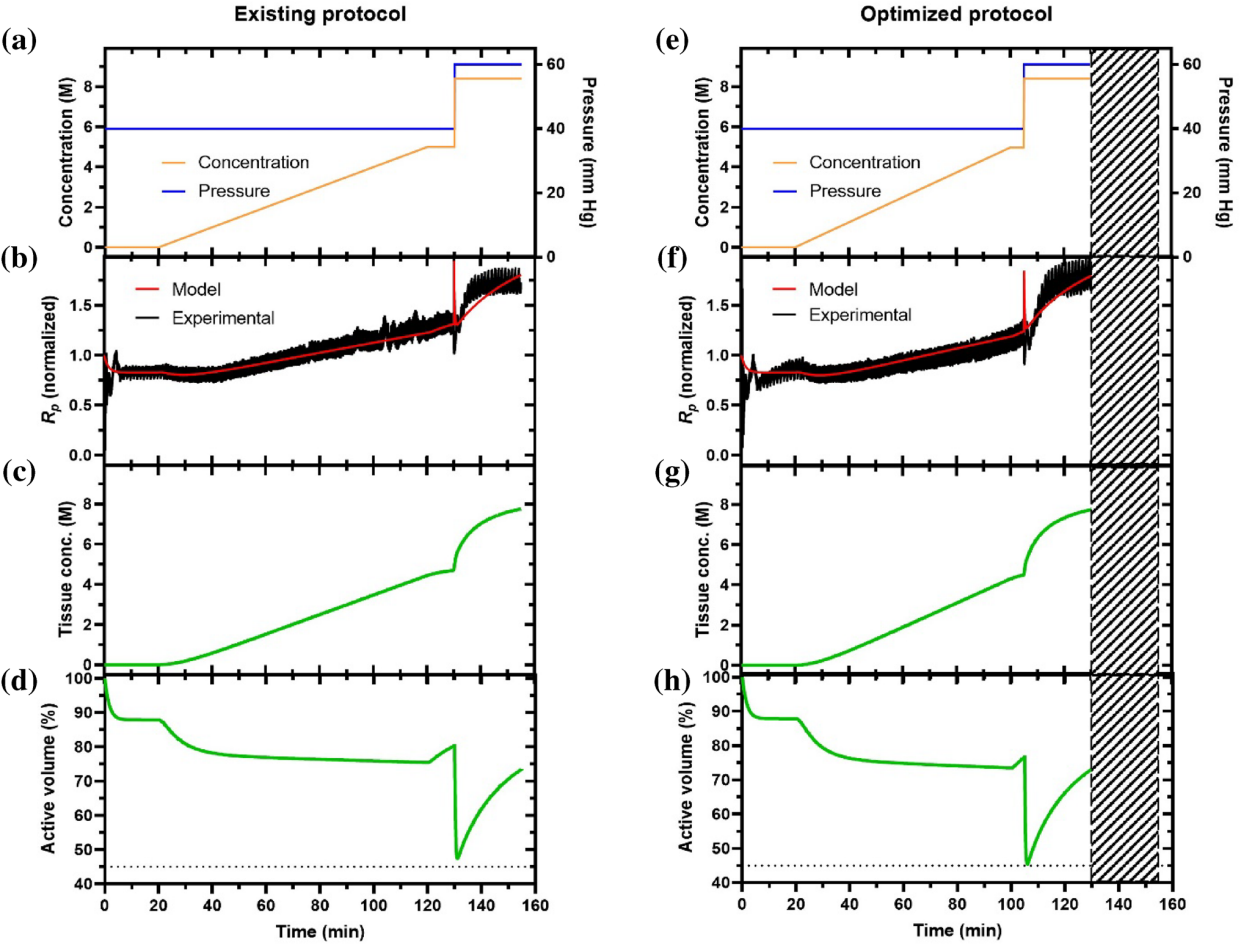
Comparison between the existing and the optimized VMP loading protocols. **a**, **e** The arterial concentration and pressure during perfusion loading. **b**, **f** The model-predicted and experimental perfusion resistances for the representative cases. *R*^2^ values equal 0.987 and 0.983 for **b** and **f**, respectively. **c**, **g** The model-predicted CPA concentration inside the kidney tissue for the representative cases. **d**, **h** The model-predicted tissue volume change for the representative cases. The dashed line represents the volume limit leading to irreversible osmotic damage, 45%. The hatched area in **e**, **f**, **g**, **h** represents time saved in the optimized protocol over the existing protocol. Data are representative cases from *n* = 3.

After obtaining the parameters in both models, we were able to predict the VMP concentration inside the kidney tissue during perfusion, predict the accumulated toxicity for the different loading protocols, and optimize the existing loading protocol. The previous loading protocol for VMP was originally proposed by Fahy et al. and modified by Han et al. into a reproducibly vitrifiable protocol using a combination of ramp and step loading, where VMP concentration was ramped from 0 to 5 M over 100 min (ramp rate = 50 mM/min), kept at 5 M for 10 min (plateau duration = 10 min), and then stepped to 8.4 M and held it for 25 min [13], as shown in Fig. 5a. The total duration for this loading protocol was 135 min. From the Krogh cylinder model, the final CPA concentration inside the kidney after loading is predicted to be 7.76 M.

In this paper, we utilized a similar ramp-hold-step pattern as the existing protocol to achieve near-optimal loading conditions while avoiding introducing osmotic damage. With more details discussed in the Supplementary material, the osmotic damage is defined by two criteria: (1) the active volume of the kidney tissues should not be below 73% of the initial active volume for an extended duration (in this paper, we assume this time to be 25 min since this is the longest time a kidney has been perfused with hypertonic CPA), and (2) the minimum active volume should not be below 45%. As the kidney shrinks below 73% of initial volume it enters a zone of osmotic stress. When volume is reduced to 45%, the kidney crosses an osmotic tolerance limit and damage occurs.

We numerically evaluated 3 parameters under this pattern: ramp rate, ramp duration, and plateau duration. The empirical ramp rates were evaluated between 40 and 70 mM/min [6, 15], which is justified as a reasonable range by the transport model, as shown in Figure S1. The empirical plateau concentration was set around 5 M, as shown in Fig. 5a [6]. Using our model, we were able to investigate increased plateau concentrations between 3 and 7 M, which allow accelerated CPA loading but must be balanced with an increase in the potential for osmotic injury. To mitigate this potential damage, we tested the ramp durations between 70 and 120 min. Additionally, we tested increased plateau durations from 4 to 20 min to allow time for osmotic equilibration. The minimum plateau duration was set to 4 min by taking some potential lagging in the practical system into consideration [39]. In summary, the ranges for parameter screening are ramp rates (40–70 mM/min), ramp duration (70–120 min), and plateau duration (4–20 min). For each tested combination of perfusion parameters, the final exposure time at full strength was calculated by the model as the value when the tissue concentration achieved 7.76 M since this is an experimentally validated vitrifiable concentration [13].

As shown in Fig. 4d, we tested ramp duration and plateau duration combinations at a given ramp rate to determine a minimized toxicity cost function. The specific simulations that support Fig. 4d for any given ramp rate between 40 and 70 mM/min are shown in Figures S2–S4. In all cases, we found that the minimum toxicity was predicted for ramp rates between 59 and 64 mM/min. Balancing the loading time reduction and potential osmotic stress, we selected the median, 62 mM/min, as the ideal ramp rate, and the two other parameters in this optimal protocol were determined to be a ramp duration of 80 min and plateau duration of 5 min, respectively. The calculated required duration at full-strength VMP is 25.7 min. The toxicity cost function value is 0.1269, suggesting the viability of the kidney after loading is predicted to be exp(− 0.1269) = 88.1%. In comparison, the existing vitrifiable loading protocol has parameters of ramp rate = 50 mM/min, ramp duration = 100 min, and plateau duration = 10 min, which has a toxicity cost function value of 0.1383, meaning the viability of the kidney after loading is predicted to be exp(˗0.1383) = 87.1%. Compared to the existing protocol, the optimized protocol has a shorter loading time (110 min vs 135 min) and lower predicted toxicity parameter (0.1269 vs. 0.1383).

Next, we tested the existing and optimized protocols experimentally and compared them to the model, as shown in Fig. 5. Both protocols include a 20-min flush with carrier solution (LM5-XZ) to stabilize the perfusion resistance. Also note that the perfusion pressures were increased to 60 mm Hg at the full-strength step to overcome the viscosity increase and maintain adequate flow rates to ensure adequate CPA transport. From Fig. 5b and f, one can tell that the experimental perfusion resistance (*R*_p_, normalized to baseline resistance established during the first 20-min of carrier solution perfusion) agrees with the model-predicted perfusion resistance very well for both loading protocols, which provides support for the validity of our model. As shown in Fig. 5c and g, the final tissue concentrations (after loading) inside the kidney are both predicted to achieve 7.76 M; this is the criteria to determine the duration of the final full-strength step. The active volume plots, Fig. 5d and h, confirmed that the active volume for both protocols did not exceed the osmotic limit (45%).

**Fig. 6:**
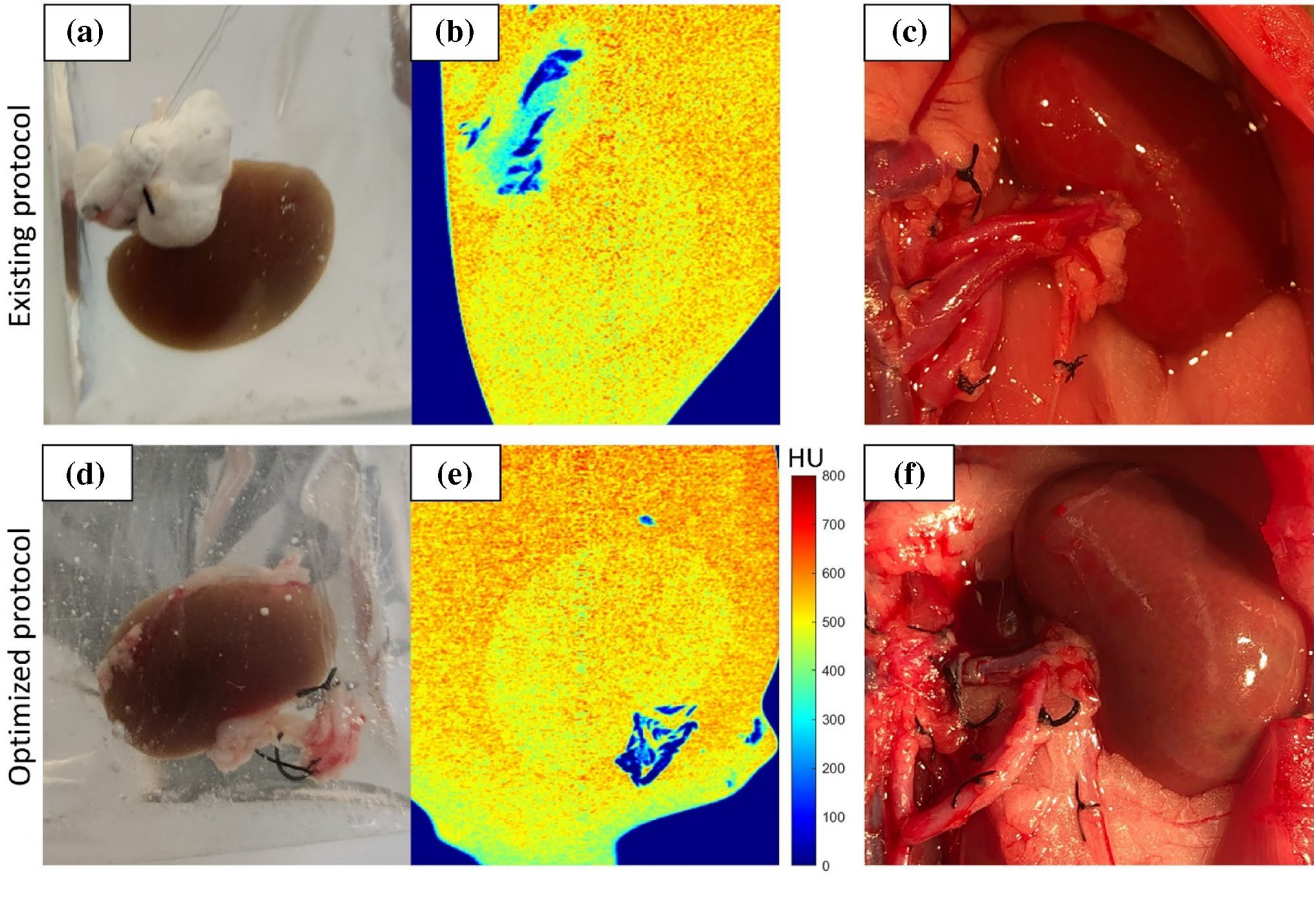
Physical (vitrification) and biological assessments of rat kidneys loaded by the two protocols. **a**, **b** Representative photo and micro-CT image of vitrified kidney perfused with the existing loading protocol. **d**, **e** Representative photo and micro-CT image of vitrified kidney perfused with the optimized loading protocol. Uniform Hounsfield units (HU) ~500 (yellow-orange) in the micro-CT images indicate vitrification. Some ice is visible on resected connective tissue (blue), but does not impact kidney viability for transplant. **c**, **f** Representative intra-operative pictures of orthotopic transplanted rat kidneys after CPA perfusion using existing and optimized protocols. Data are representative cases from *n* = 3

Furthermore, we used physical and biological assessments of the experimental kidneys to characterize the two loading protocols. First, we used a controlled rate freezer to vitrify kidneys loaded with the two protocols [39]. The photos of the two kidneys, as shown in Fig. 6a and d, suggested that both kidneys appeared to be vitrified. We then used micro-CT to validate vitrification inside the kidney, as shown in Fig. 6b and e. Based on the calibration curves of vitrified and frozen samples of different concentrations of VMP [13], both the kidneys were vitrified, and the VMP concentrations inside the kidneys were estimated at 7.83 ± 0.03 and 7.84 ± 0.03 M for the existing and optimized protocols, respectively, which are both close to the predicted value, 7.76 M. After transplantation, kidneys from both CPA protocols re-perfused immediately and uniformly after releasing vascular clamps (Fig. 6c and f), making urine within the first two minutes. The biological assessments (urine output, edema, blood gas analysis, and histology) showed that the kidneys perfused by the two protocols have equivalent results (further details are presented in Supplemental materials).

## Discussion

Long-term organ banking by vitrification would enable better donor/recipient matching, improved equity in access, better patient preparation, better transplant tolerance protocols, increased organ utilization, and enhanced graft and patient survival. To achieve this objective, development of protocols to achieve vitrifiable CPA concentrations is essential. We present an engineering approach to optimize CPA loading while minimizing toxicity in organ perfusion protocols. Based on the Krogh cylinder model, which studies CPA transport, we derived a whole organ Boyle van’t Hoff equation, extended the application of transport equations to the whole organ, and experimentally determined the relevant parameters by organ perfusion. The toxicity cost function model was adapted from previous cell and tissue work and similarly extended here to the whole organ. We selected the rat kidney as a model system to experimentally validate the developed methodology, since the kidney system has the most mature CPA perfusion protocol [13].

The existing loading protocol for VMP, designed by Fahy et al. and modified by Han et al., is predicted to achieve a viability of 87.1% after loading, which is very close to the theoretical optimum (88.1%) identified within the perfusion protocol constraints studied here. Importantly, the theoretical optimum may still be increased over what is reported here by modifying the CPA constituents or temperature during loading. The 1% theoretical difference may not be easily detected by biological assessments, confirming that the existing protocol is both reliable and feasible, supported by decades of development. Nevertheless, our approach here was able to reduce the loading protocol with VMP by 25 min and now provides the first systematic framework for developing similar protocols in other organs (e.g., heart and liver) that do not have well-developed perfusion protocols. While the 25-min reduction in time may not be essential for the kidney, since it has a cold ischemia limit of ~ 36 h, it may be critically important in other organs with shorter cold ischemia time limits, such as hearts (4 h) and livers (8–12 h) [11].

While we were able to demonstrate an effective method for describing CPA transport and toxicity behavior in organ systems, we should acknowledge several limitations of the study. All the perfusion parameters (*V*_b_/*r*_a_, *L*_p_, *ω*, and *σ*) derived and measured in this paper are essentially effective parameters representing the combined behavior of the capillary walls, cell walls, and diffusion in the extracellular space [35]. These findings reflect an averaged response for the full organ which may have regional variation based on differential anatomy and blood flow [10]. Unlike the parameters derived from cell studies or small biological systems (e.g., pancreatic islets) [42], which have direct physical meaning, the perfusion parameters are grounded in a semiempirical model that can predict the behavior of the real system. Therefore, these values are different from those obtained from the cell studies, and the values obtained from the two approaches cannot be directly applied across systems. In this paper, the CPA cocktail permeability was measured as an overall representative value; however, each component will likely permeate the membranes at different rates. For example, DMSO has a slightly larger permeability than EG in cartilage [20], but all the permeabilities of the permeants are in the same order of magnitude. Therefore, it is acceptable to regard the CPA cocktail as a single unit to estimate the permeability, and the experimental results from this paper validated this assumption.

The transport and toxicity cost function parameters for VMP were only measured at 4 °C. However, both the permeability and the toxicity rate are reduced with lower temperatures [4], so there should be an optimal temperature where the toxicity is reduced, but the permeability is still adequate to transport enough CPA into the tissue. For example, Fahy et al. used a lower temperature, − 22 °C, to decrease the toxicity of a higher concentration of CPA (M22, 9.3 M) while still having adequate CPA transport at extended times [8]. A more thorough study of the temperature dependence of toxicity and permeability would be needed to optimize the current loading protocol further. However, a similar framework could be applied, but with temperature being factored into calculations of the toxicity cost.

The optimal loading protocol design has been explored by Benson et al. for small-scale systems (e.g., oocytes) that have free boundaries [3], where during the ramping process, the oocytes were swollen and kept at the upper cell-volume limit to transport enough CPA into the cells while keeping the CPA concentration low. When the surrounding CPA concentration was suddenly changed to full-strength CPA, the oocytes’ CPA concentration rapidly increased, and the volume rapidly decreased. In organ perfusion, the idea is similar, but the tissue inside the organ cannot swell since edema will effectively reduce the vascular diameter and therefore decrease the flow and diminish CPA transport. We did not see these substantial effects of edema in our study, but this may be more important with other CPAs or tissues. In this paper, we explored the optimal loading protocol only under a ramp-hold-step pattern. We acknowledge that further improvements to the protocol design are possible, which are discussed in more detail in Supplementary material (Figures S5 and S6).

In summary, we used both models and experiments to explore mass transport and toxicity during CPA perfusion for loading and unloading in kidneys as a model system. By combining the transport kinetics and toxicity kinetics models and applying them to a rat kidney perfusion protocol, we were able to shorten the loading time by 25 min while maintaining low toxicity. For organs with shorter cold ischemia limits or higher sensitivity to established CPAs, further applications of this methodology may be particularly important.

### Supplementary Information

Below is the link to the electronic supplementary material.

Supplementary file1 (PDF 2005 kb)
