# Supplementary material for: Model-Guided Design and Optimization of CPA Perfusion Protocols for Whole Organ Cryopreservation
Source: Ann Biomed Eng. 2023 Jun 23;51(10):2216–28. doi: 10.1007/s10439-023-03255-5 (PMC10518287; doi:10.1007/s10439-023-03255-5)
Supplement: Supplementary file 1 — Supplementary file1 (PDF 2005 kb) [file 10439_2023_3255_MOESM1_ESM.pdf]

Supplementary Material

Supplementary results

Osmotic damage has been explored for small-scale systems (e.g., cells, oocytes, and sperms) [9, 11, 15], but not for large-scale systems like organs. Generally speaking, for osmotic damage occurring during cell shrinkage, cells start to show osmotic injury at ~70% of isotonic volume [2, 17], and the osmotic damage reaches an intolerable point at ~45% of isotonic volume, when the osmotic injury becomes irreversible [3, 13, 14]. In organs (e.g., kidney), a similar range can be found. Thus, most preservation solutions were designed with an osmolarity between 320 and 350 mOsm. The maximum is 400 mOsm (Sacks solution), which achieved success for short-term preservation [16]. Since isotonic osmolarity is 290 mOsm, flushing 400 mOsm preservation solution leads to the volume shrinking to 290/400 = 73% of isotonic volume. Fahy et al. also reported that the preferred tonicity for kidney perfusion is 1.2x (83% isotonic volume) to 1.5x (67% isotonic volume) [8], and they have successfully perfused M22 (1.5x tonicity, 421 mOsm) in rabbit kidneys for 25 min[7]. Moreover, it has been found that perfusing hypertonic solutions of a variety of substances (20–25% sucrose (584–730 mM)[1, 10], glucose, mannitol, glycerol, etc.) can cause renal structural damage called osmotic nephrosis, which describes a morphological pattern with vacuolization and swelling of the renal proximal tubular cells [5, 18]. In this paper, we perfused two hypertonic solutions, LM5 + 300 mM lactose solution (583 mOsm) and EC + 300 mM lactose solution (660 mOsm). We observed renal damage using EC + 300 mM lactose but not with LM5 + 300 mM lactose, suggesting that the irreversible osmotic damage happens when the volume shrinks to 44% (= 290/660) ~ 50% (= 290/583), which agrees with prior cell studies. In summary, the osmotic damage is defined by two criteria which we estimated to be: 1) the active volume of the kidney tissues should not be below 73% for extended durations (in this paper, we assume this time to be 25 min since this is the longest time a kidney has been perfused with hypertonic CPA), and 2) the minimum active volume should not be below 45%.

Under these criteria, we used the transport model and justified why the empirical ramp rate (40–70 mM/min) is reasonable, as shown in Figure S1. Here we tested a broader range of ramp rates (40–100 mM/min) to achieve 5 M prior to holding at the plateau. We then calculated the osmotic injury time which is defined as the time where the tissue cell volume is below 73%. As shown in Figure S1a, the results showed that 70 mM/min led to 22.2 min of osmotic injury time. We found that this time only increased at higher rates of loading, suggesting that the upper limit for ramp rates should be ~70 mM/min, which is in agreement with the empirical data from Fahy.

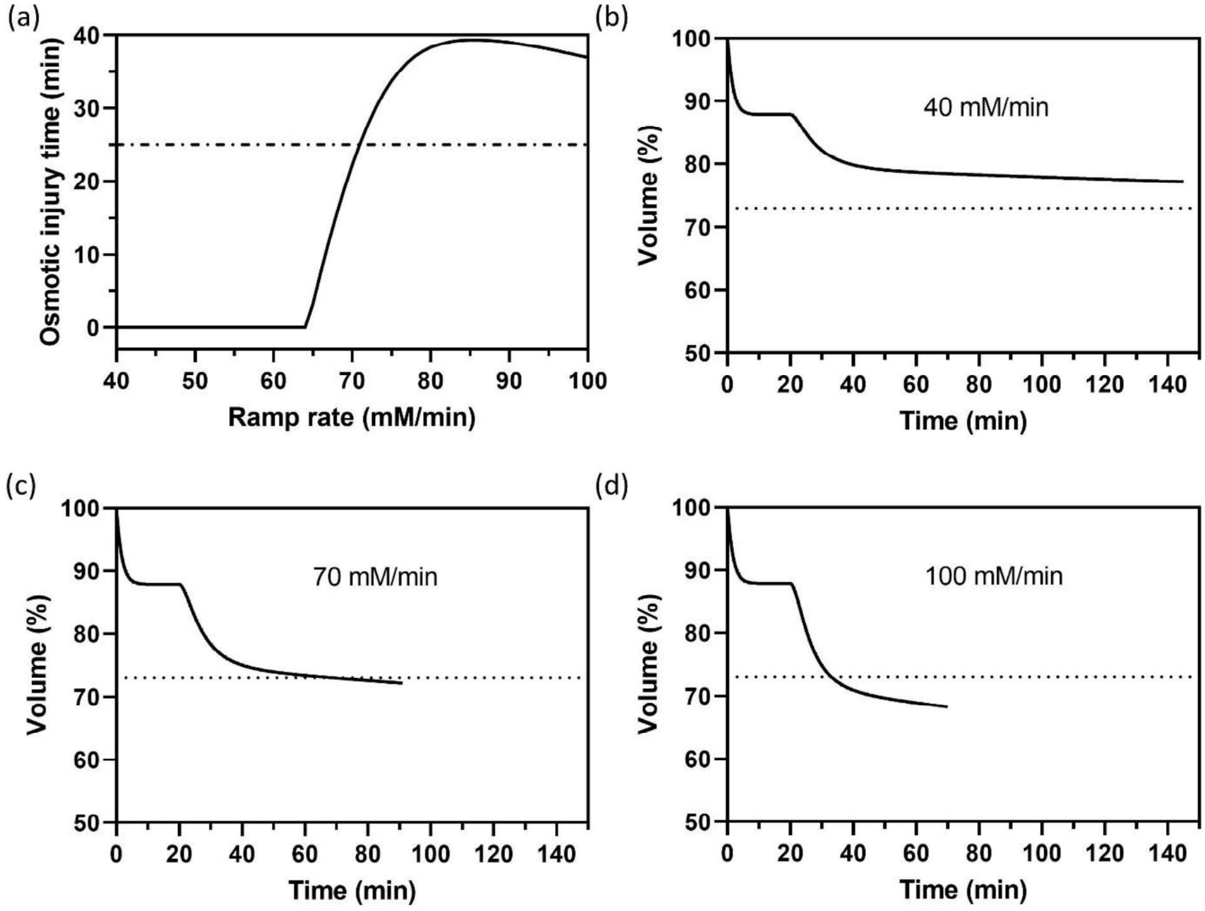

Figure S1. Osmotic injury time for different ramp rates. (a) Summary of osmotic injury time for the use of ramp rates from 40 to 100 mM/min to reach the same concentration, 5 M. The dashed line refers to the upper limit of the injury time, 25 min. (b–d) Examples of the volume change of the tissues using different ramp rates: (b) 40 mM/min, (c) 70 mM/min, and (d) 100 mM/min. The dashed line represents the volume where the osmotic injury starts, 73%.

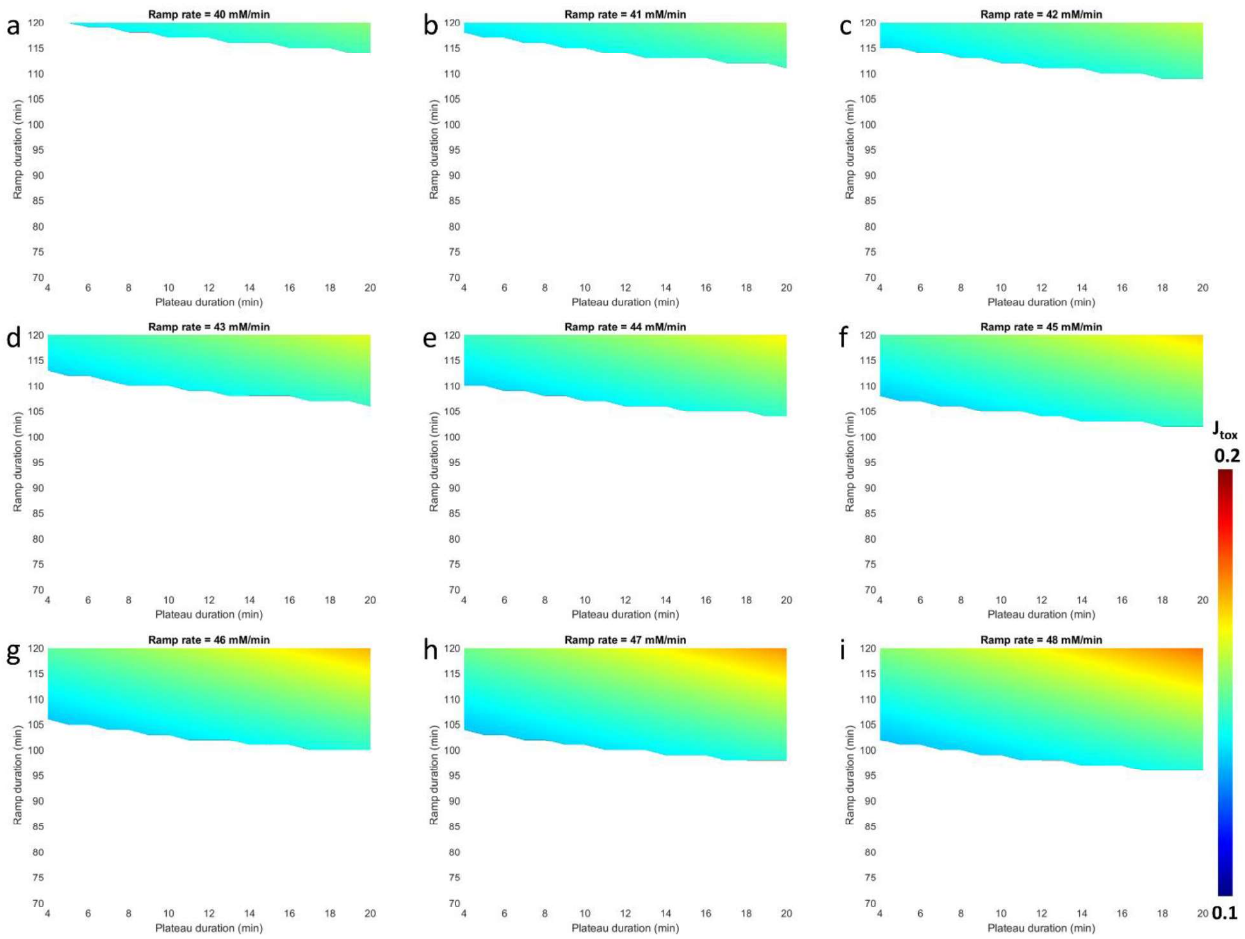

Figure S2. (a–i) Toxicity cost function values for different ramp rates, 40–48 mM/min.

Figures S2-4 show the toxicity cost function for all combinations of ramp durations and plateau durations at a given ramp rate. For small ramp rates (< 50 mM/min) with small plateau duration and small ramp duration, the tissue was not loaded with adequate CPA (displayed as white). Under these conditions the system will fail during the subsequent CPA step change, as the large osmotic shock due to the CPA bolus will shrink the tissue cell volumes below 45% and cause irreversible osmotic damage. On the other hand, for large ramp rates (>60 mM/min), the top region (displayed as white), representing long ramp durations, is also a failure mode due to osmotic injury where the tissue cell volume will be < 73% for more than 25 min. The osmotic injury time is very sensitive to ramp rate, e.g., at 65 mM/min, the minimum toxicity can be achieved using a 73-min ramp duration and 13-min plateau duration, but at 66 mM/min, the minimum toxicity can only be achieved using a 68-min ramp duration and 34-min plateau duration, which is not visible in the axis range in Figure S4i.

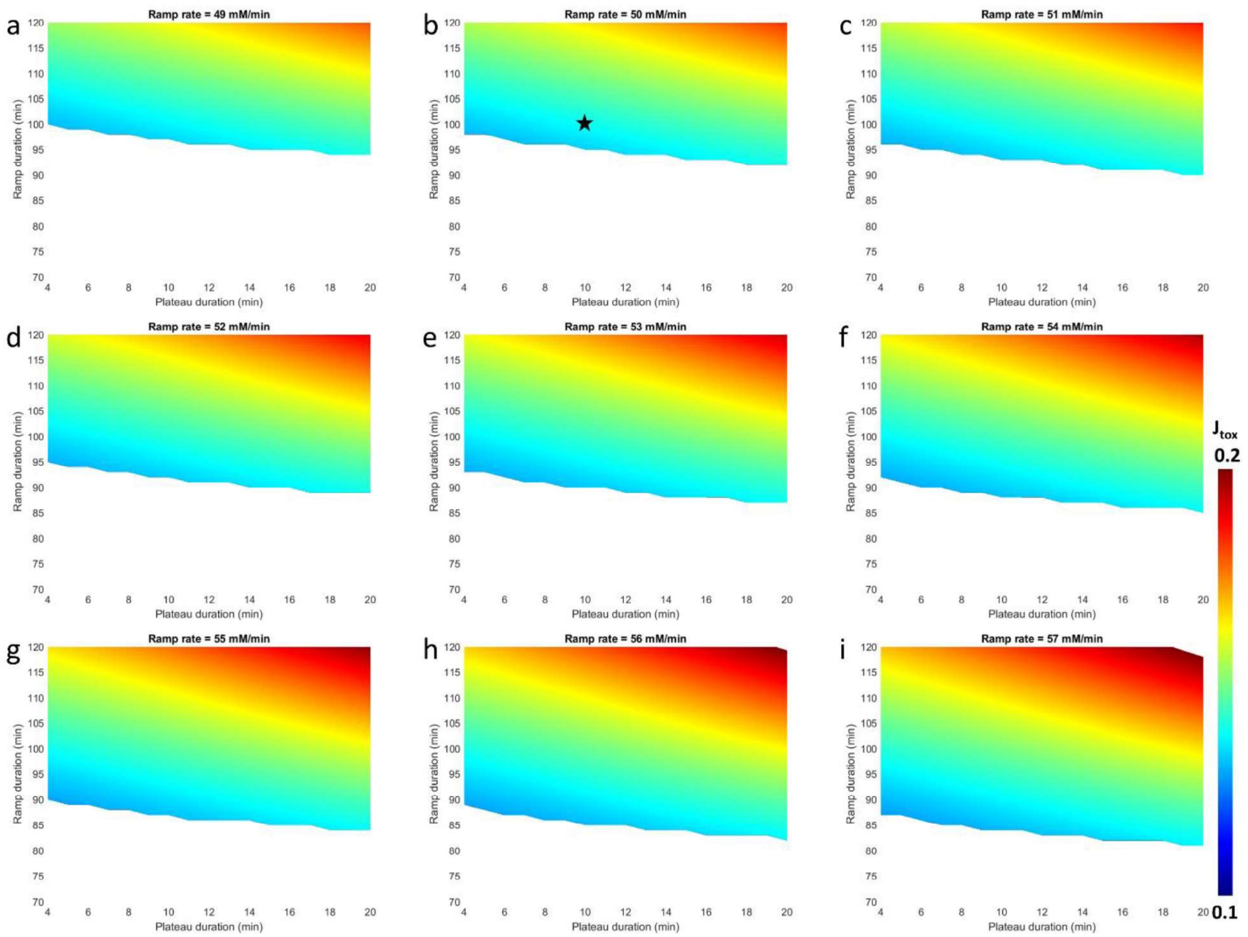

Figure S3. (a–i) Toxicity cost function values for different ramp rates, 49–57 mM/min. The solid star symbol (b) represents the existing vitrifiable VMP loading protocol.

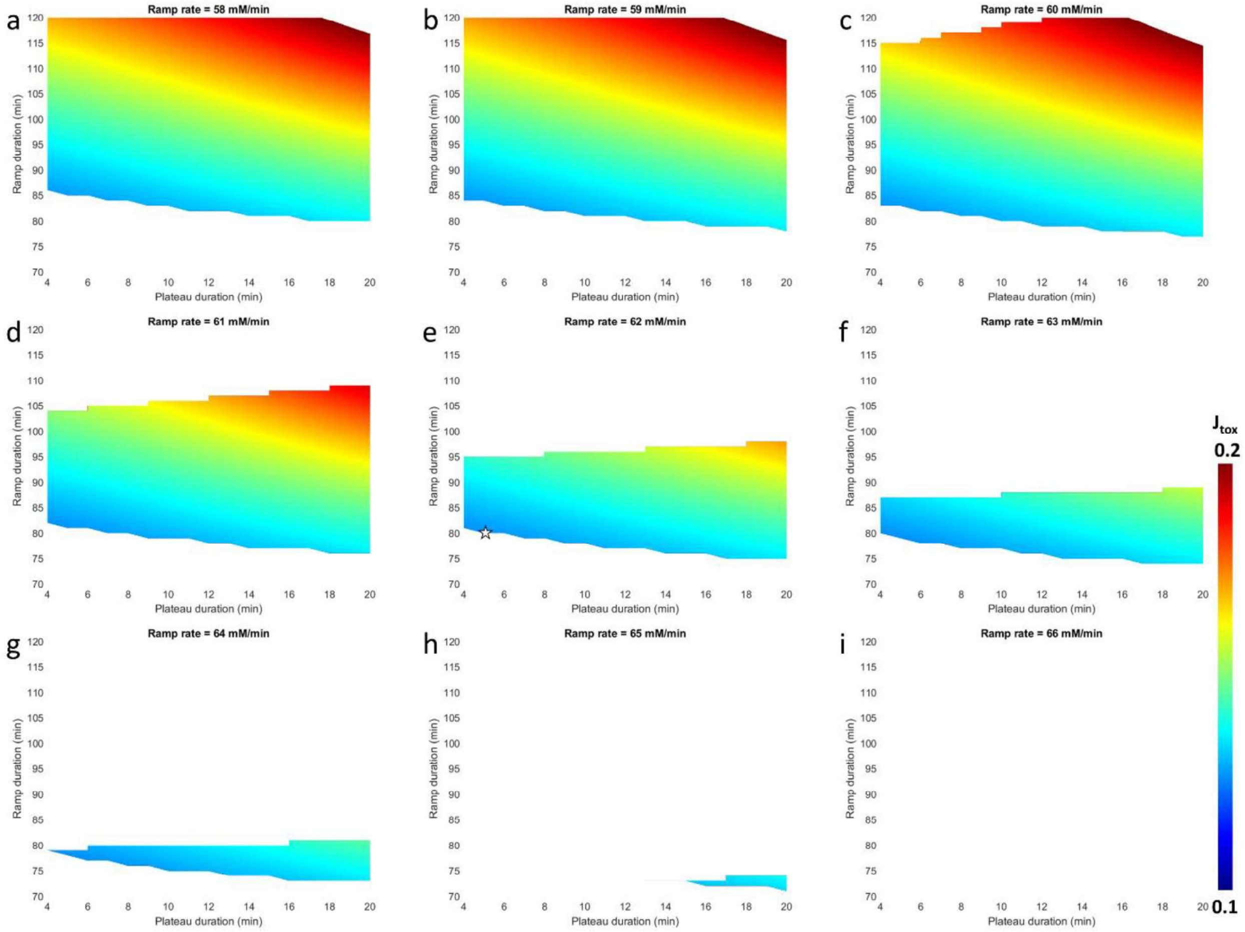

Figure S4. (a–i) Toxicity cost function values for different ramp rates, 58–66 mM/min. In Figure S4 e, the hollow star symbol represents the optimized vitrifiable VMP loading protocol.

The detailed biological assessments for the CPA-perfused kidneys using two protocols are shown in Figures S5-7. Blood gas analyses was performed every 15 minutes for a duration of 2 hours while the animal was anaesthetized. Serum potassium levels maintained within normal range throughout perfusion with the peak being 5.5mmol/l at 2-hour time point for the existing protocol and 6.4mmol/l at the 90-minute time point for the optimized protocol. The mean serum potassium levels during transplant were 3.85±0.56mmol/l and 3.85±0.76mmol/l ($P > 0.9999$) for existing protocol and optimized protocol, respectively (Fig. S5a). The peak serum sodium level in the existing protocol was 144mmol/l at the 15-minute time point when compared to 143mmol/l for the optimized protocol. The mean serum sodium was 139.36±1.99mmol/l and 140±2.23mmol/l ($P = 0.7295$) for the existing protocol and optimized protocol respectively, as shown in Figure S5b. Serum chloride levels were within normal ranges with a peak of 112mmol/l at the 75-minute mark for the existing protocol and 107mmol/l at the 60-minute time point for the optimized protocol. The mean serum chloride was 103.5±3.14mmol/l and 101.5±2.66mmol/l ($P = 0.4473$) for the existing protocol and modified protocol respectively, as shown in Figure S6c.

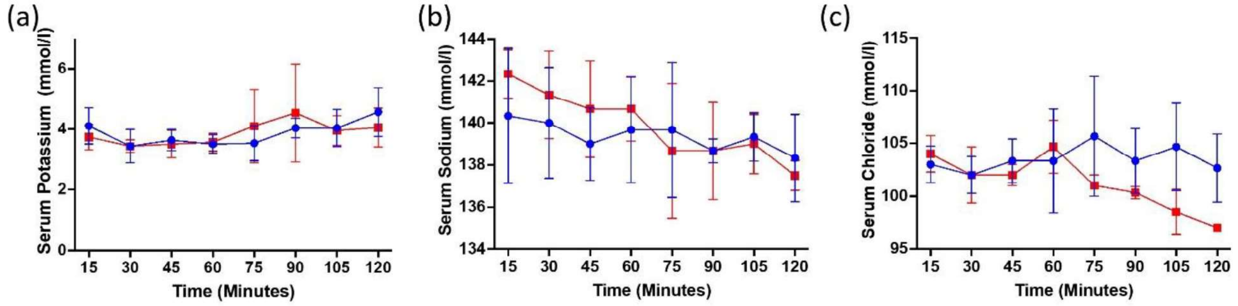

Figure S5. Serum electrolytes of transplanted VMP loaded rat kidneys. Blue lines represent existing protocol and red lines demonstrate optimized protocol of CPA loading. Blood collected from inferior vena cava every 15 minutes to measure. (n=3) (a) Sodium; (b) Potassium and (c) Chloride

Urine output was measured using the catheter placed in the donor ureter. The urine output for the existing protocol was 9.21±0.03μl/min and 8.41±0.03μl/min ($P < 0.05$) for the optimized protocol. There was no evidence of hematuria with normal color and no turbidity. Edema associated with CPA perfusion is attributed to 21.5% and 34.9% for existing protocol and optimized protocol respectively. The edema was corrected by the transplanted kidney and post-transplant edema correction was attributed to 13.8% and 20.6% for existing protocol and optimized protocol respectively. The percentage of edema after transplant to fresh control was 4.6% and 7.1% for existing protocol and optimized protocol respectively. Serum creatinine levels were maintained within normal range with the peak creatinine of 1mg/dl and 1.1mg/dl at 1 hour following reperfusion in existing protocol and optimized protocol respectively. However, the creatinine decreases in the existing protocol, while the optimized protocol continues to stay high and lowers at the 105-minute mark. The mean serum creatinine levels during the two hours of perfusion were 0.83±0.07mg/dl and 0.84±0.12mg/dl ($P = 0.9068$) for existing protocol and optimized protocol respectively, as shown in Figure S6.

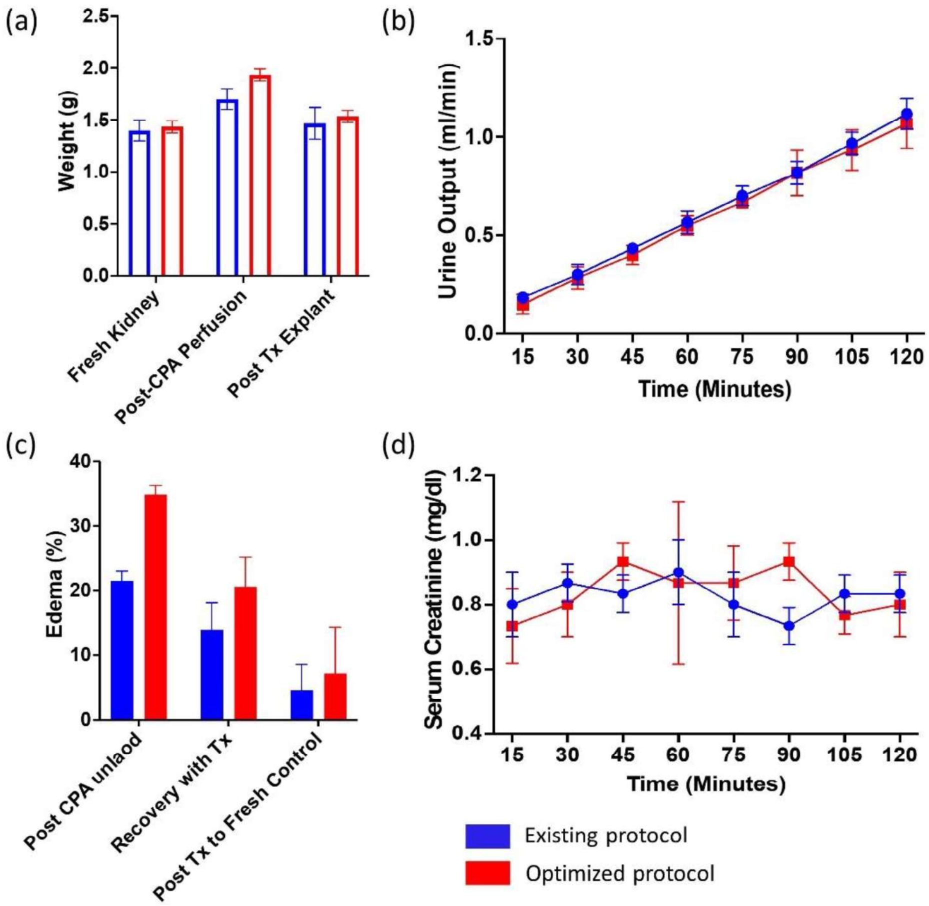

Figure S6. Edema Associated to CPA and restoration following transplant. Blue lines represent existing protocol and red lines demonstrate optimized protocol of CPA loading. (n=3) (a) Kidney weight after subsequent steps of protocol; (b). Cumulative Urine output measured from catheter every 15 minutes; (c). Edema associated with CPA and correction during transplant; (d). Serum Creatinine levels measured every 15 minutes

Transplanted kidneys from both protocols were recovered at the 2 hour time point and compared morphologically and histologically. The kidneys were bisected and both protocols demonstrated normal gross architecture in the cortex and medulla. Histological examination of the kidneys from both protocols demonstrated normal glomeruli and Bowman's space. The proximal and distal convoluted tubules show some edema which was within normal limits. There were no microthrombi in the small and large vasculature in kidneys from both protocols. Focal tubular necrosis was evident in kidneys of both protocols, but otherwise normal histology. Collecting ducts in the medulla were normal with no congestion in kidneys from both protocols, as shown in Figure S7.

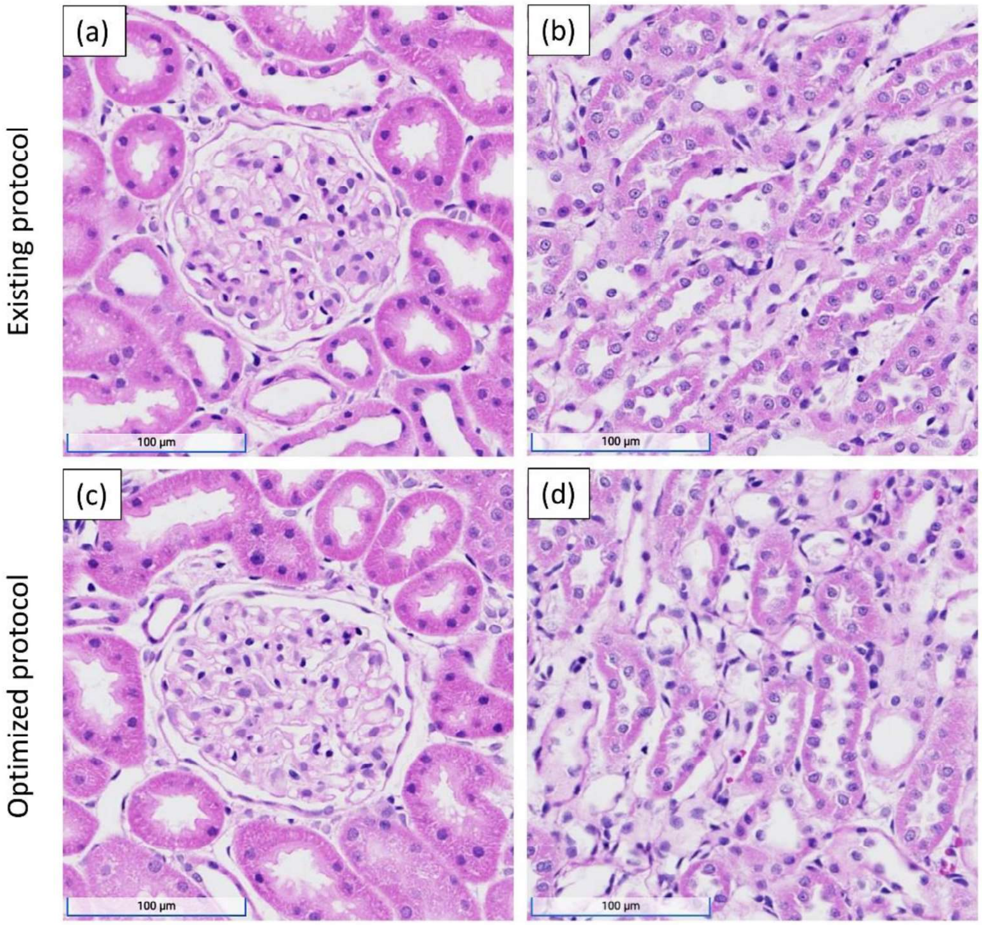

Figure S7. Rat kidney sections stained with H&E after CPA loading-unloading and transplant. (a, b). Cortex and medulla for the kidney loaded with existing protocol; (c, d). Cortex and medulla for the kidney loaded with optimized protocol. Scale bars = 100 µm.

Supplementary discussion

In this paper, we adapted ideas from small-scale systems, where osmotic injury starts to accumulate once the active volume shrinks below a value (e.g., 73%) and the osmotic injury becomes irreversible when the volume reaches a threshold (e.g., 45%). The values (73% and 45% of isotonic volume) used in this paper have not been experimentally validated in organ systems, which calls for future work. Osmotic injury should occur similarly when the tissue is swelling during the CPA unloading process, which could, if not designed properly, lead to edema and invalidate the Krogh cylinder model. In order to reduce the swelling osmotic injury, the CPA unloading protocols always add impermeants (e.g., mannitol) and apply a slower ramp, which could introduce more toxicity due to the extended exposure time. Therefore, further work is needed to explore the osmotic injury in both shrinking and swelling, which also benefits unloading protocol optimization. Moreover, lactose used in this paper may not be an ideal impermeant (σ may be less than 1). For example, the reflection coefficient for sucrose in capillaries has been estimated to be much less than 1 (0.29) [12]. Therefore, to increase accuracy of the predicted results, a

better impermeant may need to be determined or the actual reflection coefficient of lactose could be considered.

The Krogh cylinder model described the CPA and water transport inside the organs, which worked well in this paper. However, some limitations need to be acknowledged and further improvements can be considered in future work. For example, the volume (outer boundary) of the Krogh cylinder is assumed to be constant (r_k is constant) here. However, prior studies have shown that CPA perfusion can lead to substantial changes in organ volume (e.g., hearts, kidneys and brains) [4, 6, 12]. These volume changes could be taken into account to increase the accuracy of the modeling approach.

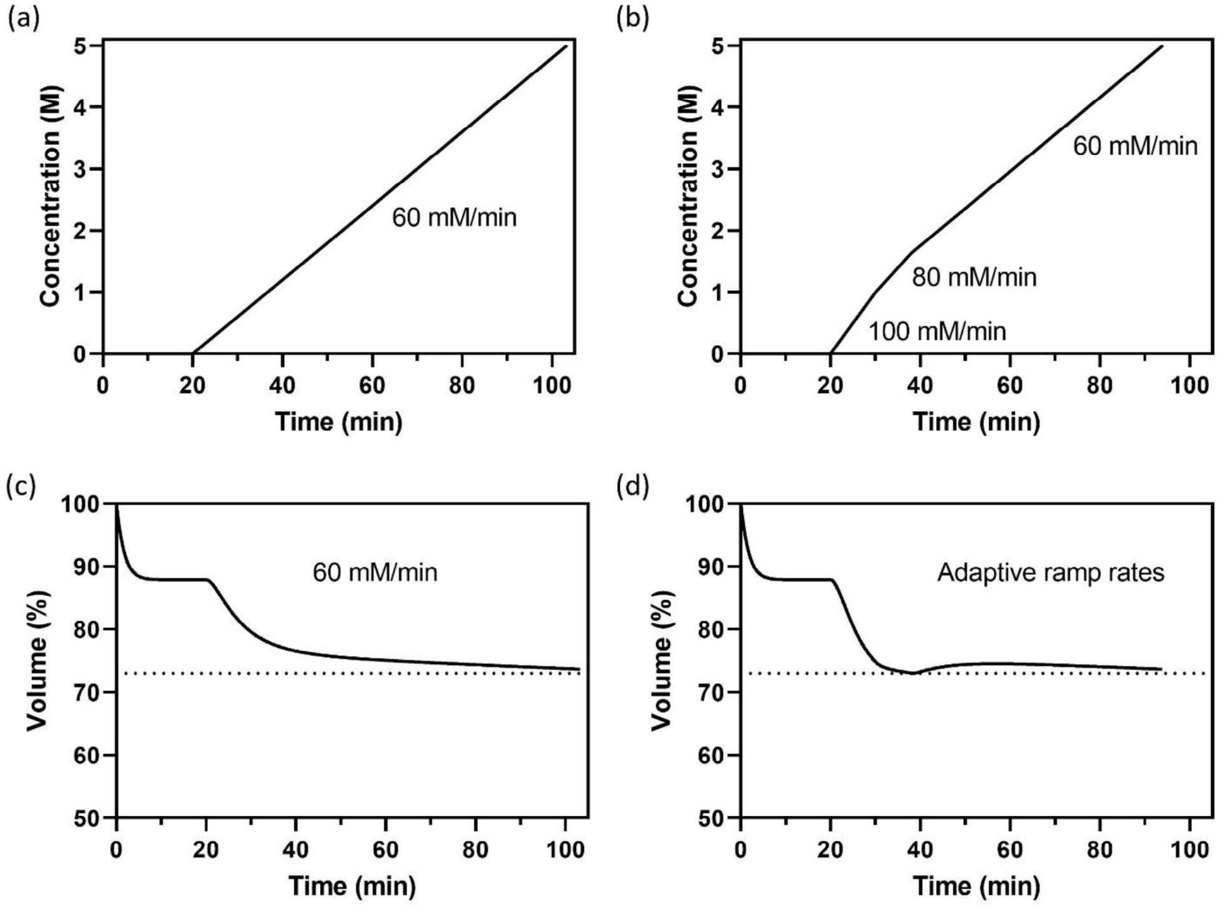

Figure S8. Comparison between the two protocols reaching the same goal concentration, 5 M. (a, c) Concentration and volume of tissue using a constant ramp rate, 60 mM/min. (b, d) Concentration and volume of tissue using an adaptive ramp rate.

We acknowledge that the optimum ramp-hold-step pattern may not be the general optimum for loading protocols. For example, we made some small modifications to a single-ramp rate protocol using adaptive ramp rates and compared two protocols ramping to the same concentration, 5 M, as shown in Figure S8. The tissue concentration for using both protocols achieved the same value, 4.345 M. For the first protocol (Figure S8a), we chose one of the ramp rates in the sweet spot, 60 mM/min. With this ramp rate, it takes 83.3 min

to reach 5 M, and the toxicity cost function value is 0.01661 after loading. For the second protocol (Figure S8b), the ramp rate changes at different times: 100 mM/min from 0 to 1 M (10 min), 80 mM/min from 1 to 1.64 M (8 min), and 60 mM/min from 1.64 to 5 M (56 min). The total loading time is 74 min, and the toxicity value is 0.01645. As shown in Figures S8 c-d, neither of the protocols will cause osmotic injury, and the second protocol reduced both the loading time (74 vs 83 min) and the toxicity (0.01654 vs 0.01661). Further optimization of the protocol design, studying the complete parameter space, will be the focus of continued work in this area.

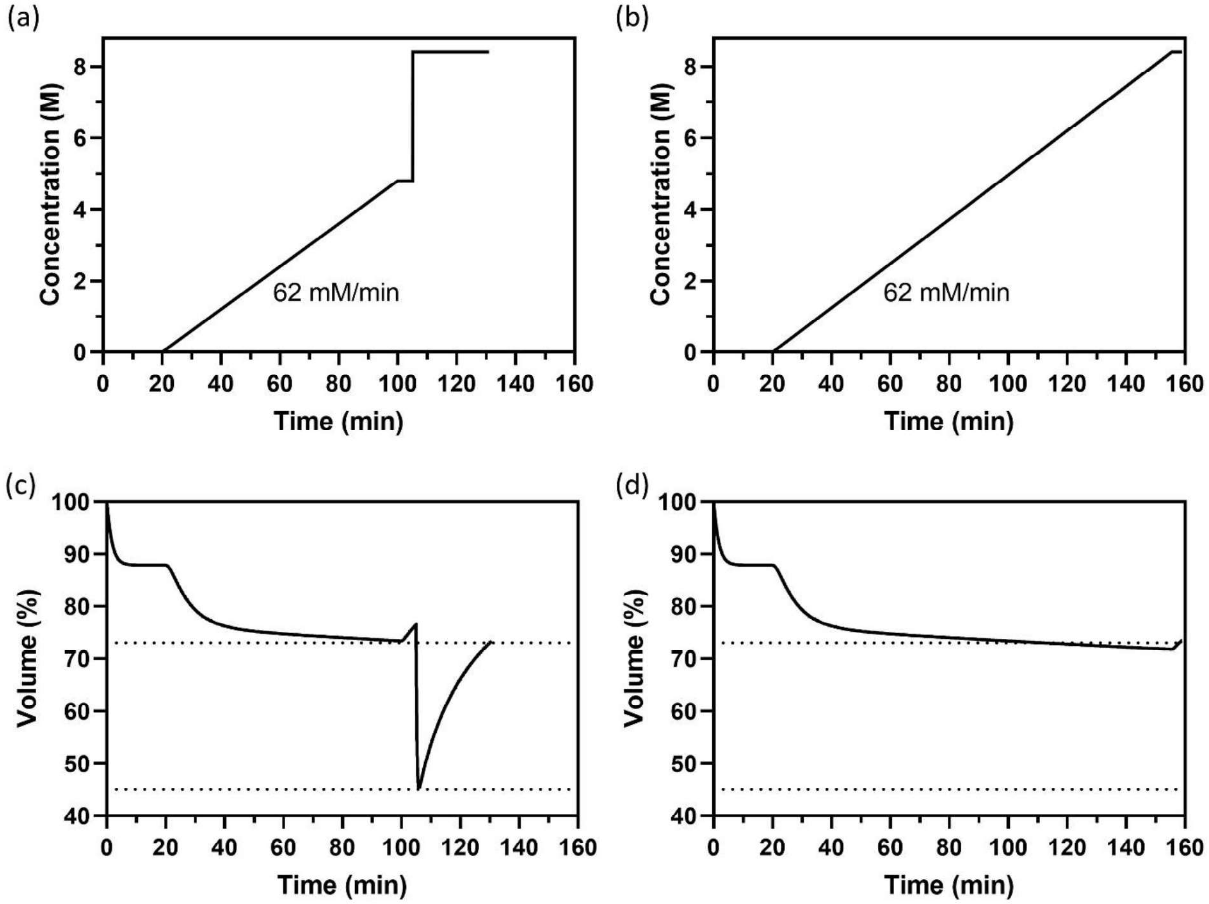

Figure S9. Comparison between using the ramp-hold-step pattern and the ramp-only pattern to achieve the same final tissue concentration, 7.76 M. (a, c) The arterial CPA concentration and tissue volume for the optimized protocol described in this paper. (b, d) The arterial CPA concentration and tissue volume for the protocol using the same ramp rate to reach the same tissue concentration, 7.76 M.

As for the advantage of using a step change, we tested two protocols with and without a step, as shown in Figure S9. The first protocol is the optimized protocol described in this paper, which has a total loading time of 110 min and a toxicity of 0.1269. The second protocol is to use the same ramp rate to reach 8.4 M during 135.5 min and hold for 3.5

min to ensure tissue concentration reaches 7.76 M. The total duration was 139 min and toxicity was 0.1837. The application of the step reduced both the loading time and the toxicity. However, whether using a steeper ramp (e.g., 200 mM/min) instead of a step from the first to the second plateau is beneficial to reducing the osmotic damage remains unknown. Further optimization can be explored in the future.
